# Special Issue “Natural Products in Drug Discovery and Development”

**DOI:** 10.3390/ijms26188762

**Published:** 2025-09-09

**Authors:** Tian-Le Gao

**Affiliations:** Institute of Materia Medica, Chinese Academy of Medical Sciences and Peking Union Medical College, Beijing 100050, China; tianlegao@imm.ac.cn

The Special Issue “Natural Products in Drug Discovery and Development” arrives at a pivotal moment when traditional medicine’s enduring wisdom is increasingly converging with modern pharmacological research. As global health challenges intensify, researchers are turning to nature’s vast repository of compounds to uncover novel therapeutic solutions. This Special Issue is designed to gather cutting-edge research articles, comprehensive reviews, and insightful case studies that explore the multifaceted potential of natural products in drug discovery and development, leveraging multidisciplinary approaches and innovative research methodologies.

A key focus lies in the isolation and characterization of natural product components. Illustrative work in this area includes the study of bile acids, which examines their bioactivities, mechanisms of action, biosynthesis, and potential applications in preventing and treating infectious diseases [1]. Several promising compounds, such as ursodeoxycholic acid, have been identified for further investigation. A notable advance is the development of artificial bear bile—produced via synthetic chemistry and enzyme engineering—which provides a sustainable and ethical alternative to natural bear bile [2]. This innovation addresses critical medical demands while aligning with animal welfare principles.

Pharmacological actions of natural compounds constitute a major research emphasis. This includes their role in ameliorating microcirculatory dysfunction induced by ischemia/reperfusion injury or lipopolysaccharide exposure, providing mechanistic insights essential for developing novel therapies targeting circulatory disorders [3]. This Special Issue further explores the role of gut microbiota in mediating drug efficacy and the beneficial effects of probiotics. For example, isochlorogenic acid C is shown to mitigate asthma through microbiota-dependent pathways [4], a finding complemented by previous research on rosmarinic acid, which modulates gut microbial composition to reduce bronchoconstriction and inflammation, offering a safer alternative to conventional asthma treatments [5]. This issue also includes a study investigating the multi-faceted benefits of berberine in improving diastolic dysfunction and mitigating metabolic comorbidities in heart failure with preserved ejection fraction (HFpEF) [6].

Innovative formulations are also highlighted, such as the development of a salt formulation combining berberine and quercetin to enhance their anti-fibrotic potential in liver fibrosis [7]. This new formulation improves the dissolution and bioavailability of quercetin, showing stronger anti-fibrotic effects than the physical mixture in both cellular and animal models. Such advancements offer new strategies for optimizing multi-drug combination therapy.

Exploring deeper biological principles, the issue aims to delve into neg-entropy mechanisms in disease treatment and prevention. Entropy, a measure of disorder, increases naturally, yet living organisms maintain their complexity and health, indicating a capacity for negative entropy [7,8]. Effective drugs may accelerate recovery by stimulating these mechanisms, which include metabolism and self-organization, defense, self-healing, wear resistance, and adaptability [8]. These interconnected capacities allow life to resist disorder, adapt to changes, and sustain complexity, providing a theoretical framework for understanding how natural products can promote health and combat disease.

The pharmacological properties of aromatic compounds and the health effects of essential oils are attracting increasing research interest. One study investigated a volatile extract from a Uyghur medicinal plant for the treatment of fluconazole-resistant vulvovaginal candidiasis [9]. The extract exhibited dual antioxidant and antifungal activities, demonstrating promising potential as a broad-spectrum natural agent against resistant fungal infections [9].

Combinational studies of natural medicine drugs are encouraged, as demonstrated by research on the synergistic analgesic effects of ligustrazine and sinomenine in treating neuropathic pain [10,11]. Network pharmacology and metabolomics analysis reveal associated targets and key pathways, supporting the clinical potential of this combination therapy [11]. Another study explores the synergistic effects of combining pregabalin with dexborneol, showing enhanced analgesic outcomes and reduced side effects, thus offering a more effective and tolerable therapeutic strategy for neuropathic pain [12].

In conclusion, this Special Issue invites readers to engage with the research and consider the transformative potential of natural medicines in advancing global healthcare. By harnessing the power of nature, we can pave the way for innovative therapies that improve human health and well-being.
